# GPR39 Suppresses Ferroptosis via the Nrf2/SLC7A11 Axis and Reduces the Sensitivity of Colorectal Cancer to Anti‐PD‐1 Immunotherapy

**DOI:** 10.1155/cjgh/5689742

**Published:** 2026-08-10

**Authors:** Cong Zhou, Xiaoling Fang, Jiaying Lin, Xiang Jiang, Huihui Li, Jiaoe Chen, Qiang Chen

**Affiliations:** ^1^ Department of Gastroenterology, Sanmen People’s Hospital, Sanmen, Taizhou 317100, Zhejiang, China, zjsmyy.com

**Keywords:** colorectal cancer, ferroptosis, GPR39, Nrf2/SLC7A11 axis, PD-1 immunotherapy

## Abstract

**Background:**

PD‐1 blockade has yet to achieve broad clinical success in colorectal cancer (CRC), with microsatellite‐stable tumors proving especially resistant. At the same time, ferroptosis has emerged as a mechanistic link between redox control in tumor cells and the antitumor immune response. GPR39 is overexpressed in CRC, but its functional role in ferroptosis and immunotherapy resistance is currently unclear.

**Methods:**

GPR39 expression was analyzed in human and mouse CRC cell lines. GPR39 knockdown, with or without the ferroptosis inhibitor liproxstatin‐1, was performed to determine whether GPR39 regulates CRC cell proliferation and migration through ferroptosis. Ferroptosis was evaluated by measuring lipid peroxidation, glutathione (GSH) levels, ferrous iron (Fe^2+^) accumulation, and reactive oxygen species (ROS). Mechanistic involvement of the nuclear factor erythroid 2–related factor 2 (Nrf2)/solute carrier family 7 member 11 (SLC7A11) signaling axis was examined using Nrf2 overexpression. Subcutaneous implantation of MC38 cells with stable GPR39 knockdown was performed to evaluate whether GPR39 regulates tumor sensitivity to anti‐PD‐1 treatment in vivo in a ferroptosis‐dependent manner.

**Results:**

GPR39 was markedly upregulated in CRC cell lines. GPR39 knockdown induced ferroptosis, characterized by increased lipid peroxidation, Fe^2+^ accumulation, and oxidative stress and accompanied by suppression of the Nrf2/SLC7A11 pathway. Nrf2 overexpression reversed these changes. Functionally, silencing GPR39 inhibited the proliferative and migratory capacities of CRC cells, effects that were largely rescued by liproxstatin‐1. In vivo, GPR39 knockdown significantly potentiated the antitumor activity of PD‐1 blockade, as evidenced by suppressed tumor progression accompanied by enhanced infiltration of CD8^+^ T cells, whereas ferroptosis inhibition abrogated these effects.

**Conclusion:**

GPR39 suppresses ferroptosis in CRC via the Nrf2/SLC7A11 axis, thereby limiting PD‐1 immunotherapy efficacy.

**Highlight**
• GPR39 is commonly upregulated in CRC cells, a finding consistent across human and mouse models.
• GPR39 depletion sensitizes CRC cells to ferroptosis through downregulation of the Nrf2/SLC7A11 axis.
• Disruption of GPR39 expression boosts the efficacy of PD‐1 blockade in CRC via ferroptosis induction.

## 1. Introduction

Colorectal cancer (CRC) represents a major global health burden, accounting for substantial cancer‐associated morbidity and mortality globally [1]. Despite ongoing improvements in surgery, chemotherapy, and radiotherapy, the clinical outcomes in advanced or metastatic CRC remain limited [2]. In recent years, immune checkpoint blockade (ICB) therapies targeting the PD‐1/PD‐L1 axis have achieved remarkable success in several solid tumors [3]. However, in CRC, durable responses induced by PD‐1/PD‐L1 inhibitors are largely restricted to patients with microsatellite instability–high tumors, whereas the vast majority of microsatellite‐stable CRC patients exhibit primary or acquired resistance [4]. These clinical observations suggest that CRC harbors strong tumor‐intrinsic resistance mechanisms that limit the effectiveness of immunotherapy, highlighting the urgent need to identify molecular determinants of immune evasion in this disease [5].

Ferroptosis, characterized by iron‐dependent lipid peroxidation, has emerged as a key process that modulating tumor metabolism, cellular homeostasis, and antitumor immunity [6]. Previous studies have confirmed that inducing ferroptosis can enhance the antitumor effect of PD‐1 blockade therapy in various malignant tumors such as melanoma [7], prostate cancer [8], gastric cancer [9], and CRC [10]. Activated CD8^+^T cells play a crucial role in this mechanism by weakening the antioxidant capacity of tumor cells, making them more susceptible to ferroptosis and immune attack [11]. Once tumor cells develop tolerance to ferroptosis, they are more likely to evade immune recognition and develop resistance to ICB [12]. These observations suggest that ferroptosis resistance represents more than a simple metabolic adaptation but a fundamental mechanism of immunotherapy failure.

G protein–coupled receptor 39 (GPR39) functions as a zinc‐sensing receptor that plays important roles in cellular stress responses [13]. Accumulating evidence has implicated GPR39 in tumor progression. GPR39 promotes proliferation, migration, invasion, and tumor growth in esophageal squamous cell carcinoma (ESCC), and its expression is elevated in CRC tissues, where it correlates with poor prognosis [14]. Notably, clinical data have identified a significant correlation between GPR39 expression levels and PD‐L1 abundance within colon cancer, suggesting a potential link between GPR39 signaling and immune escape [15]. Given that dysregulated zinc homeostasis in CRC may influence GPR39 signaling activity within the tumor microenvironment, this receptor may represent an important node connecting zinc metabolism with immune regulation [16, 17]. However, despite these observations, whether GPR39 contributes to immunotherapy resistance through regulation of ferroptosis remains unclear.

Nuclear erythroid 2–related factor 2 (Nrf2) is a core transcription factor that regulates antioxidant responses and plays an inhibitory role in ferroptosis [18]. In CRC, sustained activation of Nrf2 promotes tumor progression [19], while interference with the Nrf2/SLC7A11 pathway promotes ferroptosis and enhances the efficacy of PD‐1 blockade [20]. Previous studies have shown that GPR39 may participate in oxidative stress regulation through Nrf2 signaling. For example, activation of GPR39 with TC‐G 1008 agonist can upregulate peroxiredoxin‐1 (PRDX1) expression through Nrf2, reducing cell apoptosis caused by excessive reactive oxygen species (ROS) [21]. Our previous work also showed that activating GPR39 can improve mitochondrial function and alleviate oxidative stress through Nrf2 signaling, thereby reducing liver lipid accumulation [22]. Based on these findings, GPR39 may act as an upstream regulatory factor of the Nrf2/SLC7A11 axis, weakening the therapeutic response of CRC to PD‐1 blockade by inhibiting ferroptosis.

Therefore, this study investigates whether aberrant GPR39 activation in CRC suppresses ferroptosis via the Nrf2/SLC7A11 axis, thereby linking tumor redox regulation to resistance to PD‐1–based immunotherapy, aiming to improve the understanding of tumor‐intrinsic mechanisms underlying immunotherapy resistance in CRC.

## 2. Methods

### 2.1. Cell Culture and Treatment

Human CRC cell lines HCT116 (RRID: CVCL_0291), SW480 (RRID: CVCL_0546), and NCI‐H747 (RRID: CVCL_1587), the human normal colon epithelial cell line NCM460 (RRID: CVCL_0460), mouse CRC cell lines CT26 (RRID: CVCL_7254) and MC38 (RRID: CVCL_B288), and the mouse normal colon epithelial cell line YAMC (RRID: CVCL_6E40) were obtained from the Cell Bank of the Chinese Academy of Sciences (Shanghai, China). HCT116, SW480, NCI‐H747, CT26, and MC38 cells were cultured in high‐glucose DMEM (Gibco, USA) supplemented with 10% FBS (Gibco, USA) and 1% penicillin–streptomycin (Solarbio, USA). All cells were grown at 37°C under humidified conditions with 5% CO_2_.

For stable knockdown of GPR39, short hairpin RNA sequences designed to target human or mouse GPR39 (sh‐GPR39) and the corresponding negative control (sh‐NC) were constructed followed by packaging into lentiviral vectors by TranSheepBio (Shanghai, China). HCT116 and MC38 cells were transduced with lentivirus at a MOI of 50 with 5 μg/mL polybrene added to enhance transduction efficiency (Sigma‐Aldrich, USA). 48 h postinfection, cells were subjected to puromycin selection to generate stable GPR39 knockdown cell lines. To investigate the involvement of Nrf2 signaling, a plasmid encoding full‐length Nrf2 (oe‐Nrf2) along with the corresponding empty vector (oe‐NC) was provided by TranSheepBio (Shanghai, China). Plasmid transfection into GPR39‐knockdown cells was performed using Lipofectamine 3000 (Invitrogen, USA). Cells were collected 48 h posttransfection for subsequent molecular and functional analyses.

For ferroptosis inhibition experiments, cells were exposed to the ferroptosis inhibitor liproxstatin‐1 (50 nM; MedChemExpress, USA) for 48 h following lentiviral transduction. Control cells received an equal volume of vehicle.

### 2.2. Quantitative Real‐Time Polymerase Chain Reaction (qPCR)

Total RNA was obtained using TRIzol reagent (15596026, Invitrogen, Carlsbad, CA, USA). Reverse transcription was performed using the PrimeScript RT Reagent Kit (Takara Bio, Shiga, Japan). Quantitative real‐time PCR was carried out using TB Green Premix Ex Taq II (Takara Bio, Japan) on an ABI 7500 Fast Real‐Time PCR System (Applied Biosystems, Foster City, CA, USA). GAPDH was used as an internal control. Relative mRNA expression levels were calculated using the 2^−ΔΔCt^ method. Table 1 outlined the sequences of the primers synthesized by Tsingke Biotech Ltd.

**TABLE 1:** The primers used in the present study.

Gene	Primer (5′–3′)
Human GPR39	F: TGAGGAAGTCCGAGAGCGAAGA
	R: GCAGCCATGATCCTCCGAATCT

Mouse GPR39	F: CCCTATAACACAGGCCCAGA
	R: CACCCAAAAGAACTCCCAGA

Human SLC7A11	F: TCCTGCTTTGGCTCCATGAACG
	R: AGAGGAGTGTGCTTGCGGACAT

Mouse SLC7A11	F: CTTTGTTGCCCTCTCCTGCTTC
	R: CAGAGGAGTGTGCTTGTGGACA

Human NRF2	F: CACATCCAGTCAGAAACCAGTGG
	R: GGAATGTCTGCGCCAAAAGCTG

Mouse NRF2	F: CAGCATAGAGCAGGACATGGAG
	R: GAACAGCGGTAGTATCAGCCAG

Human GAPDH	F: GCCATCAAGGAGGCTGTAAAAGC
	R: GGTATCACCGAGGAAGTCCGTA

Mouse GAPDH	F: CATCACTGCCACCCAGAAGACTG
	R: ATGCCAGTGAGCTTCCCGTTCAG

## 3. Western Blot

Total proteins were isolated from cells or tumor samples with RIPA lysis (Beyotime, Shanghai, China) with added protease inhibitors (Roche, Basel, Switzerland). Equal protein loads (30 μg) were separated using SDS–PAGE and transferred onto polyvinylidene difluoride (PVDF) membranes (Millipore, Billerica, MA, USA). Membranes were blocked with 5% nonfat milk for 1 h at room temperature and incubated overnight at 4°C with the following primary antibodies: GPR39 (1:200, #AGR‐045‐200UL, Thermo, USA), Nrf2 (1:1000, #12721, CST, USA), SLC7A11 (1:1000, #28001, CST), and GAPDH (1:1000, #2118, CST, USA). Membranes were washed with TBST and subsequently incubated with an goat antirabbit IgG secondary antibody (HRP‐linked; 1:5000, ab6721, Abcam, UK) at RT for 1 h. Protein signals were detected with enhanced chemiluminescence detection kit, and band intensities were analyzed by ImageJ.

### 3.1. Measurement of Malondialdehyde (MDA) and Glutathione (GSH) Levels

To assess oxidative lipid damage and intracellular antioxidant capacity, MDA and GSH levels were measured with commercially available assay kits (Elabscience, Wuhan, China). Briefly, cells were harvested and subjected to lysis with the lysis buffer provided in the kit, and subsequently centrifuged at 12,000 × g for 10 min at 4°C. The resulting supernatants were collected. For tumor tissues, samples were weighed, homogenized in the supplied extraction buffer on ice, and centrifuged under the same conditions. MDA level was evaluated based on the thiobarbituric acid reaction, and absorbance was recorded at 532 nm. Total GSH content was determined with an enzymatic recycling method, with absorbance recorded at 405 nm. All absorbance values were detected with microplate reader. MDA and GSH concentrations were determined according to standard curves generated in parallel and normalized to BCA‐measured total protein.

### 3.2. Detection of Intracellular Ferrous Iron (Fe^2+^)

Intracellular Fe^2+^ content was evaluated with the FerroOrange fluorescent probe (#36104, CST). Briefly, cells were seeded on coverslips followed by two washes with HBSS, followed by exposure to FerroOrange working solution diluted in HBSS at 37°C in the dark for 30 min. After staining, cells were washed gently to remove excess probe. Fluorescence signals corresponding to intracellular Fe^2+^ were captured using a fluorescence microscope (Leica DMi8, Wetzlar, Germany) under identical exposure and gain settings for all experimental groups.

### 3.3. Detection of Lipid ROS

Lipid ROS production was assessed with a lipid peroxidation–responsive fluorescent probe C11‐BODIPY probe (581/591; Invitrogen, USA). Cells were exposed to C11‐BODIPY working solution diluted in medium (serum‐free) for 30 min at 37°C under dark conditions and washed with phosphate‐buffered saline (PBS). Images were obtained with a fluorescence microscope under identical imaging parameters. For quantitative analysis, fluorescence intensities of the oxidized (green) and nonoxidized (red) signals were measured using ImageJ software.

### 3.4. Colony Formation Assay

Long‐term colony‐forming ability was assessed by seeding cells at low density (approximately 500–1000 cells/well) in six‐well plates and culturing them for 10–14 days under standard conditions. During this period, medium changes were performed every 3‐4 days. Following incubation, cells were fixed with 4% paraformaldehyde (15 min) and stained with 0.1% crystal violet (20 min). Only colonies containing > 50 cells were included in the analysis and counted manually.

### 3.5. Cell Viability Assay

Cell viability was assessed by a CCK‐8‐based method (Dojindo, Japan). Cells were seeded into 96‐well plates at an appropriate density and followed by overnight attachment. At designated time points, the CCK‐8 solution was applied to individual wells and followed by incubation at 37°C for 1‐2 h. Optical density was measured at 450 nm with the BioTek Synergy H1 microplate reader (Agilent, USA) to determine relative cell viability.

### 3.6. Transwell Migration Assay

Cell migration ability was evaluated with transwell inserts with porous membranes (Corning, USA). Cells were resuspended in serum‐deprived medium and seeded into the upper chamber, while medium containing 10% fetal bovine serum was added to the lower chamber as a chemoattractant. After incubation at 37°C for 24 h, nonmigrated cells on the upper surface of the membrane were removed. Migrated cells on the lower surface were fixed with 4% paraformaldehyde, stained with crystal violet, and counted in randomly selected fields using light microscopy.

### 3.7. Animal Model and Treatment

Male C57BL/6 mice aged 6–8 weeks were supplied by Vital River Laboratory Animal Technology Co., Ltd. (Beijing, China) and kept under SPF conditions with free access to food and water maintained on a 12 h light/dark cycle. Each experimental group consisted of six mice (*n* = 6). MC38 cells stably transduced sh‐NC or sh‐GPR39 were harvested during the logarithmic growth phase, resuspended in PBS, and subcutaneously inoculated into the right axillary subcutaneous region at a density of 1 × 10^6^ cells/mouse. Tumor volume was assessed at 2–3 day intervals with a digital caliper. When tumor grew to around 50 to 100 mm^3^, mice were randomly assigned to different groups. Anti‐PD‐1 antibody (200 μg/mouse; Bio X Cell, USA) or isotype IgG control was administered by intraperitoneal injection every 3 days. The ferroptosis inhibitor liproxstatin‐1 (10 mg/kg; MedChemExpress, USA) or vehicle control was administered intraperitoneally once daily. Treatments were continued for 28 days, after which animals were humanely euthanized, and tumors were resected, weighed, and processed for further analyses. All animal experiments were approved by the Institutional Animal Care and Use Committee of Sanmen People’s Hospital and were conducted in accordance with institutional guidelines and the ARRIVE guidelines.

### 3.8. Dihydroethidium (DHE) Staining

ROS accumulation in tumor tissues was evaluated via DHE‐based assay. Fresh specimens were first embedded in OCT and cryosectioned (8–10 μm). Next, the tissue sections were incubated with DHE working solution (37°C, 30 min, in the dark) and rinsed with PBS. Fluorescence signals, indicating intracellular ROS levels, were captured using a Leica DMi8 fluorescence microscope. All groups were imaged under the same exposure conditions.

### 3.9. Immunofluorescence Staining

Tumor tissues were collected and fixed in 4% paraformaldehyde for 24 h. The specimens were then dehydrated through a graded ethanol series. After dehydration, the tissues were embedded in paraffin blocks. These blocks were subsequently sectioned. The section thickness was set to 5 μm. The paraffin sections were first deparaffinized and then rehydrated using a graded alcohol series. To expose the antigens, the sections were heated in citrate buffer. The pH of this buffer was 6.0. Following antigen retrieval, the sections were blocked. The blocking solution was 5% BSA. After blocking, the sections were incubated with the primary antibody. The antibody used was anti‐CD8 (1:100, #sc‐1177, Santa Cruz, USA). This incubation was carried out overnight at 4°C. The next day, the sections were washed to remove unbound antibody and then incubated with a secondary antibody. This antibody was goat antimouse IgG (H&L) conjugated to Alexa Fluor 488. It was diluted at 1:500 and purchased from Abcam. Incubation with the secondary antibody lasted for 1 h at RT. To visualize cell nuclei, the sections were stained with DAPI. Finally, all stained sections were examined. Fluorescence images were captured using a microscope (Leica DMi8, Germany). For the quantitative analysis, multiple fields were selected at random. All images for analysis were acquired under identical imaging settings.

### 3.10. Immunohistochemistry (IHC) Staining

The tumor tissue used in the experiment was embedded in paraffin and prepared into 4‐μm‐thick slices. We slice and perform routine dewaxing and rehydration, and then heat in citrate buffer for 15 min to complete antigen retrieval. Endogenous peroxidase was blocked with 3% H_2_O_2_, and nonspecific binding was blocked with 5% BSA at room temperature for 30 min. The sections were incubated with anti‐Ki67 antibody (1:200, ab16667, Abcam, UK), incubated overnight at 4°C. The sections were incubated with an HRP‐conjugated secondary antibody (1:3000, ab6721, Abcam, UK) for 60 min at room temperature. Then, the sections were subjected to DAB staining, hematoxylin counterstaining, dehydration, and sealing, followed by image acquisition under an optical microscope. When quantifying, we randomly select multiple fields of view under the same imaging conditions and count Ki67 positive nuclei.

### 3.11. Statistical Analysis

Each experiment was repeated a minimum of three times. Results were shown as mean ± SD. GraphPad Prism 10.1.2 was used for all statistical evaluations. Student’s *t*‐test was employed to compare two groups. For comparisons involving more than two groups, one‐way ANOVA with Tukey’s post hoc test was applied. Statistical significance was defined as *p* < 0.05.

## 4. Results

### 4.1. GPR39 Is Upregulated in Colorectal Cancer Cell Lines

**FIGURE 1:**
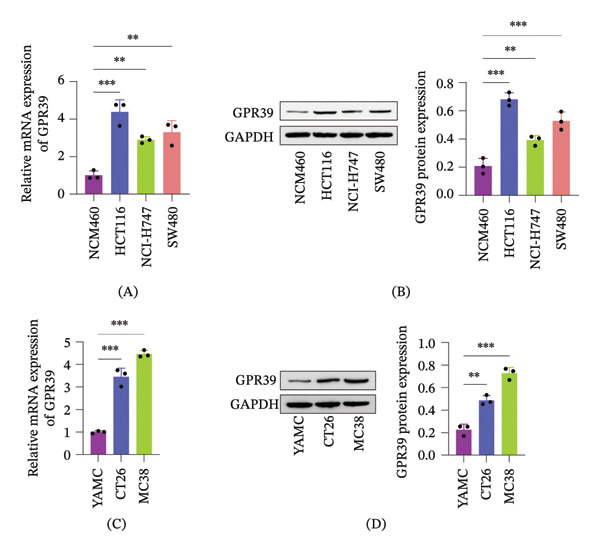
GPR39 is upregulated in human and mouse colorectal cancer cell lines. (A) Relative mRNA expression levels of GPR39 in human colorectal cancer cell lines (HCT116, SW480, and NCI‐H747) and the human normal colon epithelial cell line NCM460, as determined by qPCR. (B) Protein expression levels of GPR39 in human colorectal cancer cell lines (HCT116, SW480, and NCI‐H747) and the human normal colon epithelial cell line NCM460, as determined by Western blot analysis. (C) Relative mRNA expression levels of GPR39 in mouse colorectal cancer cell lines (CT26 and MC38) and the mouse normal colon epithelial cell line YAMC, as determined by qPCR. (D) Protein expression levels of GPR39 in mouse colorectal cancer cell lines (CT26 and MC38) and the mouse normal colon epithelial cell line YAMC, as determined by Western blot analysis. Data were exhibited as means ± SD from three independent experiments (*n* = 3). ∗∗*p*  <  0.01, ∗∗∗*p* <  0.001.

To establish the expression profile of GPR39 in CRC, we evaluated its mRNA and protein levels. Multiple CRC cell lines of both human and mouse origin were examined. qPCR and western blot analyses showed that GPR39 expression was markedly increased in human CRC cell lines (HCT116, SW480, and NCI‐H747) versus the normal human colon epithelial cell line (NCM460) (Figure 1A,B). Consistently, GPR39 mRNA and protein abundance were substantially increased in murine CRC cell lines (CT26 and MC38) relative to YAMC mouse colon epithelial cells (Figure 1C,D). Among the tested cell lines, GPR39 expression was relatively higher in HCT116 (human) and MC38 (mouse). Therefore, HCT116 and MC38 cells were selected for subsequent functional and mechanistic experiments.

### 4.2. GPR39 Knockdown Induces Ferroptosis and Suppresses the Nrf2/SLC7A11 Pathway in CRC Cells

To determine whether GPR39 participates in the regulation of ferroptosis in CRC, stable GPR39 knockdown was established in HCT116 and MC38 cells using shRNA. The most efficient knockdown shRNA was selected for subsequent experiments (sh‐GPR39‐1 for HCT116 and sh‐GPR39‐2 for MC38) (Figure 2A,B). The effects of GPR39 silencing on ferroptosis‐related biochemical and molecular markers were then evaluated. As shown in Figure 2C,D, knockdown of GPR39 led to a marked elevation of MDA levels, concomitant with a pronounced reduction in GSH levels in both HCT116 and MC38 cell models. Consistently, fluorescence probe–based assays revealed that GPR39 silencing led to a pronounced increase in intracellular Fe^2+^ levels, as indicated by enhanced FerroOrange fluorescence intensity (Figure 2E). In addition, C11‐BODIPY 581/591 staining showed an increase in green fluorescence in both cell lines following GPR39 knockdown, indicating elevated lipid peroxidation and lipid ROS accumulation (Figure 2F). At the molecular level, western blot and qPCR analyses revealed that knockdown of GPR39 markedly downregulated the abundance of Nrf2 and its regulated target SLC7A11 in HCT116 and MC38 cells (Figure 2G,H). To further clarify the involvement of Nrf2 signaling, Nrf2 was overexpressed in GPR39‐silenced cells. It was observed that the overexpression of Nrf2 led to the restoration of SLC7A11 expression at the protein level (Figure 2I). In addition, this overexpression of Nrf2 markedly suppressed the accumulation of lipid ROS (Figure 2J). Collectively, these results indicate that the knockdown of GPR39 promotes ferroptosis in CRC cells. This process appears to be mediated, at least in part, through the suppression of the Nrf2/SLC7A11 signaling axis.

FIGURE 2Knockdown of GPR39 induces ferroptosis and suppresses the Nrf2/SLC7A11 pathway in colorectal cancer cells. (A–B) Protein expression levels of GPR39 in HCT116 and MC38 determined by Western blot; (C) intracellular MDA levels in HCT116 and MC38 cells following GPR39 knockdown, measured using commercial assay kits; (D) intracellular GSH levels in HCT116 and MC38 cells following GPR39 knockdown, measured using commercial assay kits; (E) intracellular Fe2^+^ levels detected by the fluorescent probe FerroOrange in control and GPR39‐silenced cells; (F) lipid ROS levels detected by C11‐BODIPY 581/591 staining in HCT116 and MC38 cells after GPR39 knockdown; (G) protein expression levels of Nrf2 and SLC7A11 analyzed by Western blot in control and GPR39 knockdown cells; (H) mRNA expression levels of Nrf2 and SLC7A11 determined by qPCR analysis in control and GPR39‐silenced cells; (I) protein expression levels of Nrf2 and SLC7A11 determined by Western blot analysis in GPR39‐silenced cells with or without Nrf2 overexpression; (J) lipid ROS levels assessed by C11‐BODIPY 581/591 staining in GPR39 knockdown cells following Nrf2 overexpression. Data were exhibited as means ± SD from three independent experiments (*n* = 3). ∗∗*p* <  0.01, ∗∗∗*p* <  0.001.

**Figure:**
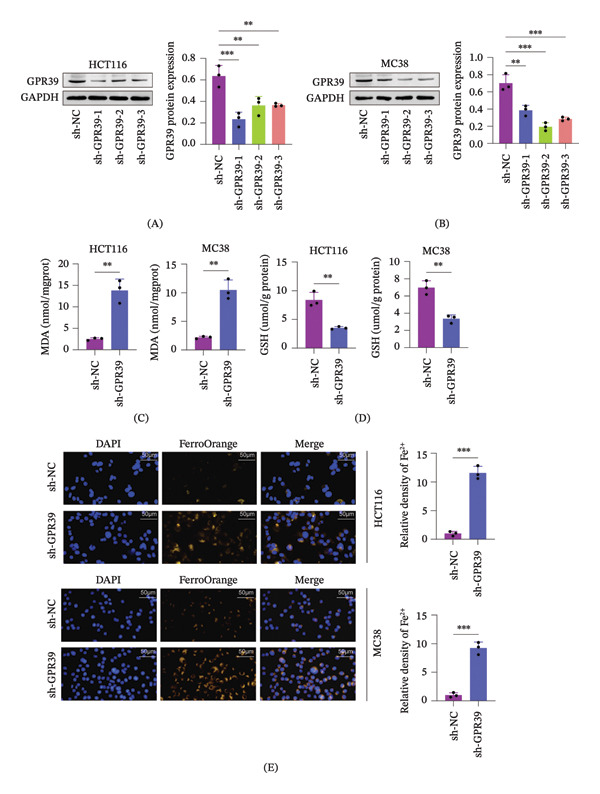

**Figure:**
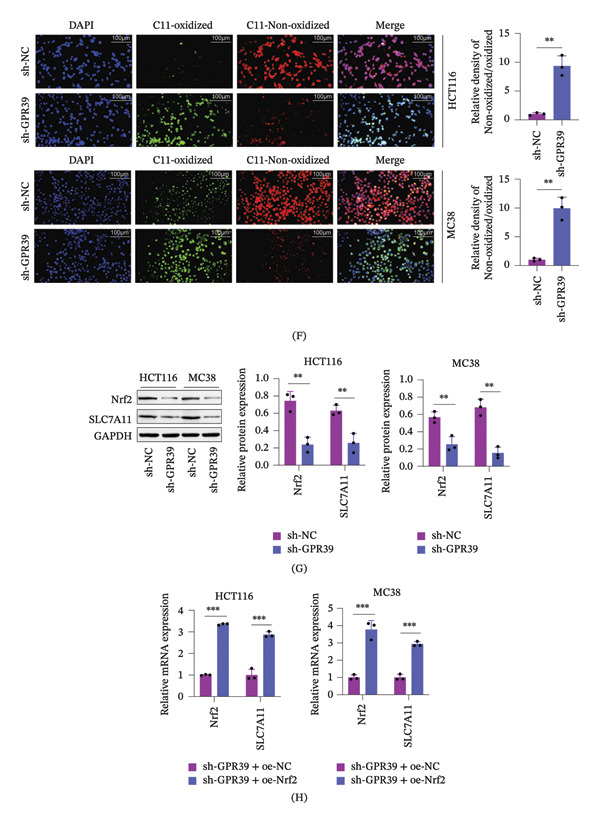

**Figure:**
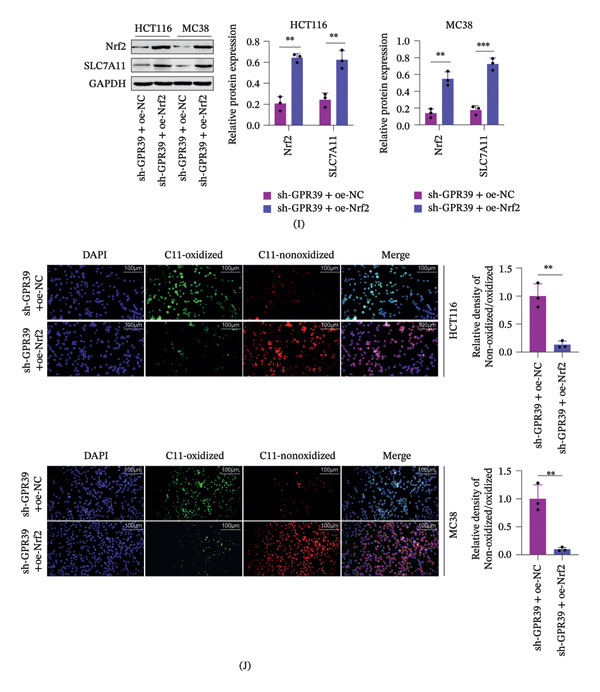

### 4.3. Liproxstatin‐1 Reverses the Inhibitory Effects of GPR39 Knockdown on CRC Cell Proliferation and Migration

**FIGURE 3:**
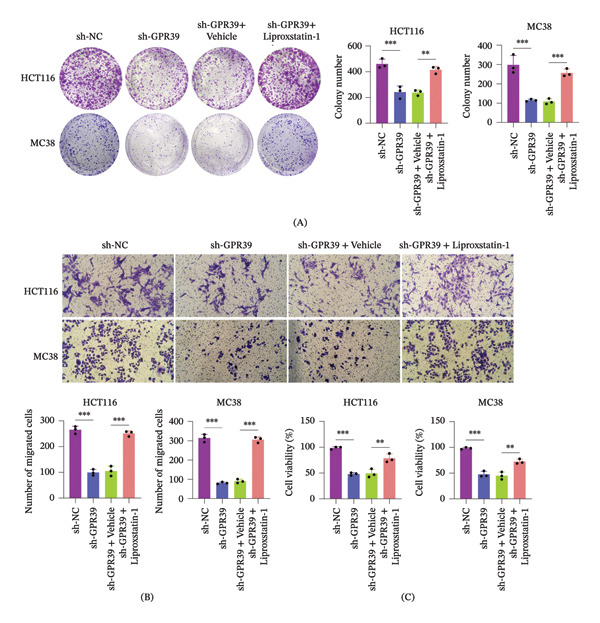
Liproxstatin‐1 reverses the inhibitory effects of GPR39 knockdown on colorectal cancer cell proliferation and migration. (A) Colony formation assays assessing the proliferative capacity of control and GPR39‐silenced CRC cells after liproxstatin‐1 treatment; (B) transwell migration assays assessing the migratory ability of control and GPR39‐silenced CRC cells after liproxstatin‐1 treatment; (C) CCK‐8 assays assessing cell viability in control and GPR39‐silenced CRC cells after liproxstatin‐1 treatment. Data were exhibited as means ± SD from three independent experiments (*n* = 3). ∗∗*p* <  0.01, ∗∗∗*p* <  0.001.

To clarify whether ferroptosis mediates the effect of GPR39 on the proliferation and migration of CRCs, we conducted a series of functional experiments. Firstly, GPR39 was stably knocked down in HCT116 and MC38 cells, followed by treatment with the ferroptosis‐specific inhibitor liproxstatin‐1, and cell proliferation, migration, and survival abilities were detected separately. The results showed that after knocking down GPR39, the colony‐forming ability (Figure 3A) and transwell migration number of both cell lines decreased (Figure 3B), and CCK‐8 detection also showed a significant decrease in cell viability rate (Figure 3C). On the contrary, the inhibitory effects on proliferation, migration, and survival were significantly reversed after the addition of liproxstatin‐1. These results indicate that the effect of GPR39 on the proliferation and migration of CRCs depends on ferroptosis signaling.

### 4.4. GPR39 Knockdown Increases CRC Responsiveness to Anti‐PD‐1 Therapy by Promoting Ferroptosis In Vivo

**FIGURE 4:**
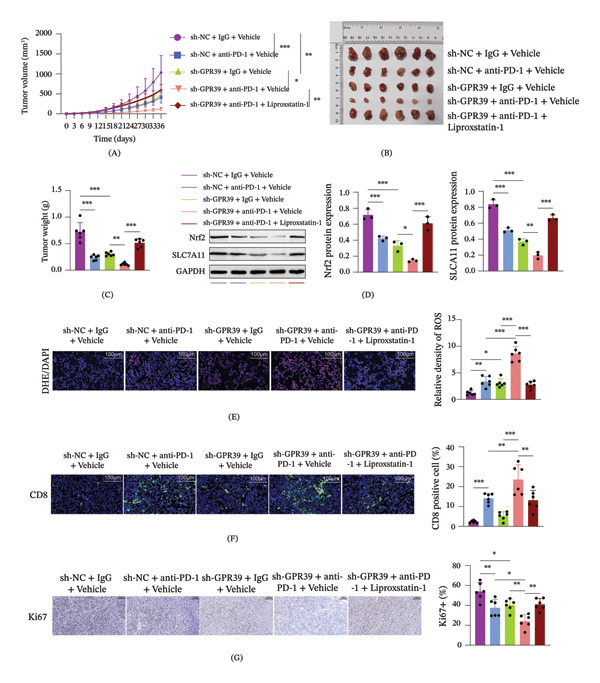
Knockdown of GPR39 enhances the sensitivity of colorectal cancer to anti‐PD‐1 therapy by promoting ferroptosis in vivo. (A) Tumor growth curves of MC38 syngeneic tumors under the indicated treatment conditions; (B) representative images of excised tumors at the experimental endpoint; (C) tumor weights measured at the end of the experiment; (D) protein expression levels of Nrf2 and SLC7A11 in tumor tissues analyzed by Western blot; (E) ROS levels in tumor tissues detected by DHE staining; (F) immunofluorescence staining showing CD8^+^ T‐cell infiltration in tumor tissues under the indicated conditions. (G) immunohistochemical staining of Ki67 in tumor tissues under the indicated conditions. Each group consisted of six mice (*n* = 6). Data were exhibited as mean ± SD. ∗*p* <  0.05, ∗∗*p* <  0.01, ∗∗∗*p* <  0.001.

To evaluate the in vivo function of GPR39 in CRC and its impact on the therapeutic response to anti‐PD‐1 immunotherapy, MC38 cells with or without stable GPR39 knockdown were subcutaneously injected into the right axilla of C57BL/6 mice to establish syngeneic tumor models. When tumor volumes reached approximately 50–100 mm^3^, mice were treated as indicated, and the experiment was terminated on Day 28. As shown in Figure 4A–C, compared with the sh‐NC + IgG + vehicle group, treatment with anti‐PD‐1 antibody alone or GPR39 knockdown alone resulted in moderate inhibition of tumor growth. Notably, compared with the sh‐GPR39 + IgG + vehicle group, combined GPR39 knockdown and anti‐PD‐1 antibody treatment markedly enhanced the antitumor effect, leading to significant suppression of tumor growth. In contrast, administration of the ferroptosis inhibitor liproxstatin‐1 substantially attenuated the therapeutic response to anti‐PD‐1 treatment in GPR39‐knockdown tumors, indicating reduced sensitivity to anti‐PD‐1 treatment. To further assess ferroptosis‐related signaling in tumor tissues, the expression of Nrf2 and SLC7A11 and the level of ROS were analyzed. As shown in Figure 4D,E, anti‐PD‐1 antibody treatment alone or GPR39 knockdown alone slightly suppressed the Nrf2/SLC7A11 axis and increased ROS levels. In contrast, GPR39 knockdown combined with anti‐PD‐1 therapy significantly downregulated the expression of Nrf2 and SLC7A11, accompanied by a significant increase in ROS levels, which can be reversed by the ferroptosis inhibitor liproxstatin‐1. In terms of immune cell infiltration, the combination therapy group showed a moderate increase in CD8^+^T cell infiltration in tumor tissue (Figure 4F); Meanwhile, the number of Ki67‐positive tumor cells was further reduced compared to using anti‐PD‐1 alone or knocking out GPR39 alone (Figure 4G). After adding liproxstatin‐1, the opposite trend was observed: an increase in Ki67 positive cells and a decrease in CD8^+^T cell infiltration. In summary, knocking out GPR39 can enhance the sensitivity of CRC to PD‐1 therapy by promoting ferroptosis, thereby inhibiting tumor growth in vivo.

## 5. Discussion

Immunotherapy based on PD‐1 blockade has shown long‐lasting clinical benefits, which have been demonstrated in various malignant tumors. However, the efficacy of this type of therapy in CRC is still limited, especially in microsatellite‐stable tumors. In this study, we identified GPR39 as a regulatory factor of ferroptosis and revealed that it regulates the sensitivity of CRC to PD‐1 blockade through the Nrf2/SLC7A11 signaling axis. The above findings provide important new insights for the treatment of CRC.

GPR39 is a zinc ion–sensitive GPCR that participates in regulating cellular adaptation processes to metabolism and oxidative stress [13]. In recent years, its function in tumor biology has received attention, especially in tumor growth and invasion. For example, in ESCC, overexpression of GPR39 promotes cell proliferation and migration [23]. In CRC, elevated expression levels are associated with poor prognosis [23]. In clinical practice, there is a positive correlation between GPR39 and PD‐L1 expression in CRC tissues, suggesting that they may be involved in immune escape [15]. We also observed sustained high expression of GPR39 in multiple human and mouse CRC cell lines, which further suggests that it may be a stress‐related oncogenic factor. Based on these findings, upregulation of GPR39 expression in CRC may represent a mechanism by which tumors enhance cellular adaptability and cope with treatment pressure during progression.

Notably, our previous work in hepatocyte models demonstrated that GPR39 regulates cellular redox homeostasis through the SIRT1/Nrf2 signaling axis, highlighting the ability of GPR39 to modulate antioxidant stress responses through downstream signaling pathways [22]. Beyond SIRT1, accumulating evidence indicates that GPR39 can engage multiple downstream signaling pathways, including PI3K/AKT and MAPK signaling, to regulate diverse biological processes such as oxidative stress adaptation, cell viability, proliferation, and tissue repair in a context‐dependent manner [24]. Collectively, these findings suggest that GPR39 functions as a multifunctional signaling hub rather than acting through a single downstream pathway, providing a mechanistic basis for its broad regulatory roles in tumor biology. Our study further expands the biological significance of GPR39 by linking its redox‐regulatory function to ferroptosis and immunotherapy response through the Nrf2/SLC7A11 axis in colorectal cancer.

The Nrf2/SLC7A11 signaling axis constitutes a central endogenous defense system against ferroptosis by sustaining antioxidant capacity and limiting lipid peroxidation, and its hyperactivation has been broadly associated with ferroptosis resistance, tumor aggressiveness, and therapeutic failure across multiple cancers [25]. Previous studies have primarily focused on intracellular regulators of this axis, such as KEAP1 mutations or metabolic reprogramming, whereas the contribution of upstream membrane receptors has remained largely unexplored [26]. In this context, our findings identify GPR39 as an upstream regulator of Nrf2/SLC7A11 signaling in CRC, extending earlier observations that GPR39 alleviates oxidative stress through Nrf2‐dependent pathways in nonmalignant tissues to a malignant and immunologically relevant setting. Importantly, our functional rescue experiments place these molecular observations into a causal framework. While prior studies have shown that genetic or pharmacological inhibition of Nrf2 or SLC7A11 enhances ferroptosis sensitivity and suppresses tumor growth [27], our data demonstrate that modulation of a GPCR is sufficient to reprogram ferroptotic susceptibility and associated malignant behaviors. Suppression of GPR39 lowers the ferroptosis threshold, resulting in impaired proliferative and migratory capacities. The ability of liproxstatin‐1 to restore proliferative and migratory capacities further distinguishes ferroptosis‐driven functional impairment from nonspecific cytotoxicity. Rather than reflecting generalized growth arrest, the observed phenotypes suggest a ferroptosis‐dependent loss of adaptive resilience, in which lipid peroxidation and iron‐dependent oxidative stress constrain malignant behavior. This interpretation is particularly relevant in the tumor‐associated microenvironment, in which malignant cells are continuously exposed to oxidative pressure imposed by immune effector cells [28]. Taken together, these findings indicate that ferroptosis may influence malignant cellular behaviors and that GPR39‐mediated regulation of ferroptotic susceptibility is associated with the maintenance of proliferative and migratory potential in CRC cells under oxidative stress.

Recent immuno‐oncology studies have established ferroptosis as a critical interface between tumor‐intrinsic redox regulation and antitumor immunity [29]. Tumor‐associated macrophages produce itaconate, which is taken up by tumor cells via the SLC13A3 transporter to activate the NRF2‐SLC7A11 pathway, thereby conferring ferroptosis resistance and diminishing the efficacy of ICB [20]. Our in vivo findings extend this framework by identifying GPR39 as a ferroptosis‐suppressive factor that constrains PD‐1 therapeutic efficacy in CRC. While either GPR39 knockdown or PD‐1 blockade alone exerts only modest antitumor activity, their combination produces pronounced tumor suppression, accompanied by increased oxidative stress, reduced proliferative activity, and enhanced CD8^+^ T‐cell infiltration. The ability of liproxstatin‐1 to abrogate these effects provides functional evidence that ferroptosis is a key mediator of the observed therapeutic synergy, rather than a secondary consequence of immune activation. Notably, these results are consistent with emerging reports in melanoma [7], gastric cancer [9], and prostate cancer [8], where sensitization to ferroptosis augments responsiveness to immune checkpoint inhibitors. Our study extends these observations to CRC and further reveals a tumor‐intrinsic GPCR–Nrf2–SLC7A11 signaling axis as a previously unrecognized mechanism of immune resistance. Conceptually, this work supports a model in which modulation of ferroptosis thresholds determines whether immune pressure is translated into effective tumor control, indicating that targeting ferroptosis resistance could constitute a viable strategy to sensitize immunologically “cold” CRC tumors to PD‐1‐based immunotherapy.

Of course, we are also aware that there are some limitations to the research. First, the experimental system is currently mainly based on cell lines and animal models, and further validation is needed in CRC samples from patients in the future. Second, we mainly analyzed the regulatory mechanism of ferroptosis in tumor cells themselves, but whether GPR39 affects the interaction between tumors and immune cells through this process still needs further investigation. Third, although we focused on the Nrf2/SLC7A11 pathway, the regulatory network of ferroptosis in CRC is actually more complex and involves multiple metabolic and redox‐related pathways that were not fully explored in this study. Therefore, if more metabolic and redox‐related regulatory factors can be included in future research, it will help to better understand how GPR39 affects ferroptosis resistance and immunotherapy. Fourth, the upstream signaling mechanism by which GPR39 activates the Nrf2/SLC7A11 axis in colorectal cancer remains incompletely understood. Whether known downstream signaling pathways of GPR39, such as SIRT1 or PI3K/AKT, mediate this process warrants further investigation. Fifth, canonical ferroptosis markers, including GPX4 and ACSL4, were not evaluated in this present study. Nevertheless, ferroptosis was assessed using multiple complementary biochemical and functional assays that are widely used for ferroptosis evaluation, including GSH depletion, MDA accumulation, Fe^2+^ accumulation, lipid ROS generation, and ferroptosis inhibitor rescue [30]. Future studies incorporating GPX4 and ACSL4 will provide more comprehensive mechanistic validation. Sixth, although increased CD8^+^ T‐cell infiltration was observed, the present study did not comprehensively characterize the functional state of the tumor immune microenvironment. Future studies incorporating analyses of effector cytokines (e.g., IFN‐*γ* and granzyme B), PD‐L1 expression, and immunosuppressive cell populations, including regulatory T cells and myeloid‐derived suppressor cells, will provide deeper mechanistic insight into how GPR39‐mediated ferroptosis modulates the response to anti‐PD‐1 immunotherapy.

## 6. Conclusions

This study indicates that GPR39 is involved in the regulation of ferroptosis in colorectal cancer through the Nrf2/SLC7A11 axis and correlates with reduced sensitivity to PD‐1‐based immunotherapy.

## Author Contributions

Cong Zhou, Huihui Li, and Jiaoe Chen guaranteed the integrity of the entire study. Cong Zhou and Xiang Jiang designed the study and literature research. Xiaoling Fang and Jiaying Lin defined the intellectual content. Cong Zhou, Xiaoling Fang, and Jiaoe Chen performed experiment. Xiaoling Fang, Xiang Jiang, and Qiang Chen collected the data. Jiaying Lin, Huihui Li, and Jiaoe Chen analyzed the data. Cong Zhou, Xiaoling Fang, and Qiang Chen wrote the main manuscript and prepared figures. All authors reviewed the manuscript.

## Funding

This work was supported by the Zhejiang Medical and Health Science and Technology Plan (grant number: 2024XY092).

## Ethics Statement

All animal experiments were approved by the Institutional Animal Care and Use Committee of Sanmen People’s Hospital and were conducted in accordance with institutional guidelines and the ARRIVE guidelines.

## Consent

The authors have nothing to report.

## Conflicts of Interest

The authors declare no conflicts of interest.

## Data Availability

The data that support the findings of this study are available on request from the corresponding author. The data are not publicly available due to privacy or ethical restrictions.
